# Nanoscale microenvironment engineering based on layer-by-layer self-assembly to regulate hair follicle stem cell fate for regenerative medicine

**DOI:** 10.7150/thno.48723

**Published:** 2020-09-22

**Authors:** Peng Chen, Yong Miao, Feifei Zhang, Junfei Huang, Yuxin Chen, Zhexiang Fan, Lunan Yang, Jin Wang, Zhiqi Hu

**Affiliations:** Department of Plastic and Aesthetic Surgery, Nanfang Hospital, Southern Medical University, 1838 North Guangzhou Avenue, Guangzhou, Guangdong 510515, China.

**Keywords:** hair follicle stem cells, layer-by-layer self-assembly, stem cell microenvironment, tissue engineering, regenerative medicine

## Abstract

Hair regenerative medicine, a promising strategy for the treatment of hair loss, will likely involve the transplantation of autologous hair follicular stem cells (HFSCs) and dermal papilla cells (DPCs) into regions of hair loss. Cyclic hair regeneration results from the periodic partial activation of HFSCs. However, previous studies have not successfully achieved large-scale HFSC expansion *in vitro* without the use of feeder cells*,* with a lack of research focused on regulating HFSC fate for hair follicular (HF) regeneration. Hence, an emerging focus in regenerative medicine is the reconstruction of natural extracellular matrix (ECM) regulatory characteristics using biomaterials to generate cellular microenvironments for expanding stem cells and directing their fate for tissue regeneration.

**Methods:** HFSCs were coated with gelatin and alginate using layer-by-layer (LbL) self-assembly technology to construct biomimetic ECM for HFSCs; after which transforming growth factor (TGF)-β2 was loaded into the coating layer, which served as a sustained-release signal molecule to regulate the fate of HFSCs both *in vitro* and *in vivo*. *In vitro* experiments (cell culture and siRNA) were employed to investigate the molecular mechanisms involved and *in vivo* implantation was carried out to evaluate hair induction efficiency.

**Results:** Nanoscale biomimetic ECM was constructed for individual HFSCs, which allowed for the stable amplification of HFSCs and maintenance of their stem cell properties. TGF-β2 loading into the coating layer induced transformation of CD34^+^ stem cells into highly proliferating Lgr5^+^ stem cells, similar to the partial activation of HFSCs in HF regeneration. Thus, LbL coating and TGF-β2 loading partially reconstructed the quiescent and activated states, respectively, of stem cells during HF regeneration, thereby mimicking the microenvironment that regulates stem cell fate for tissue regeneration during HF cycling. Improved HF regeneration was achieved when the two HFSC states were co-transplanted with neonatal mouse dermal cells into nude mice.

**Conclusion:** This study provides novel methods for the construction of stem cell microenvironments and experimental models of HF regeneration for the treatment of hair loss.

## Introduction

Hair loss is a common disorder in humans and is generally caused by genetics, aging, diseases, and drugs 1. Although not life-threatening, hair loss has social and psychological implications, and thus, an increased demand for its treatment has emerged 2. Current treatment options include various medications and autologous hair transplantation; however, they remain limited by the inability to regenerate new hair follicles (HFs) 3,4. Hence, tissue engineering of HFs has an important clinical significance for the treatment of hair loss 5.

HF reconstruction requires sufficient hair-inducing seed cells, represented by hair follicle stem cells (HFSCs) as well as dermal papilla cells (DPCs) 5. Although strides have been made toward improving *in vitro* amplification of DPCs and maintaining their hair inductivity 6-11, advances in HFSCs continue to face certain challenges 12-15. Stem cells reside in spatially distinct microenvironments termed niches that consist of neighboring cells, extracellular matrix (ECM) and signals 16. *In vitro* stem cells abandon the niche microenvironments and begin to lose their stem cell properties 17,18. Hence, the recent advances made in tissue engineering and regenerative medicine exploring biomimetic biomaterials to mimic stem cell microenvironments and amplify multipotent HFSCs, has become a promising potential solution.

Layer-by-layer (LbL) self-assembly is a thin-film fabrication technique for coating cells that functions by depositing multilayer-coatings of oppositely charged polycation and polyanion materials on cell surfaces 19,20. LbL differs from other techniques that encapsulate cells into microscale hydrogels, which generally yield high polymer-to-cell ratios and lack control over the hydrogel's mechanical properties 21. LbL self-assembly is a single-cell nanoscale surface modification technique that offers unique advantages with an extensive range of biomedical applications, including controlled drug delivery 22, biosensors 23, targeted gene therapy 24, and tissue engineering 25,26. Multilayered films using polysaccharides and proteins are considered a promising approach for engineering nanoscale biomimetic cellular microenvironments 27. For instance, alginate is a natural polysaccharide derived from algae, and gelatin is a protein derivative. Owing to their biocompatibility and biodegradability, these substances have been widely used in ECM tissue engineering 28-32. In the current study, gelatin and alginate were used to coat HFSCs in the engineering of nanoscale biomimetic ECM for individual cells.

Hair growth is a cyclical process consisting of three phases, growth (anagen), degeneration (catagen), and rest (telogen) 33. During the transition from late telogen to early anagen, quiescent HFSCs are partially activated by signals from the dermal papilla (DP), resulting in the conversion of niche quiescent CD34^+^ HFSCs to activated, high proliferating, leucine-rich repeat-containing G-protein-coupled receptor 5^+^ (Lgr5^+^) HFSCs, which ultimately form new HFs 34,35. Previous studies have found that androgenic alopecia is associated with stem cell activation disorders, while the number of quiescent stem cells in the niche remains unchanged 36. Lgr5^+^ stem cells can differentiate into all cell lineages found in the hair structure, including the hair shaft and inner root sheath. Hence, the knockout of Lgr5^+^ stem cells prevents hair from entering the growth phase, suggesting that these cells are essential for HF regeneration 34. Furthermore, transplantation studies demonstrate that Lgr5^+^ cells are the most potent population of cells for regenerating HFs 37. Therefore, not only should critical seed cells (HFSCs) be expanded for HF regeneration, but cells with hair inductivity should also be constructed. However, to date no *in vitro* studies have successfully induced the conversion of CD34^+^ stem cells to Lgr5^+^ stem cells for promoting HF regeneration.

Nevertheless, LbL self-assembly technology has proven effective for use in regulating cellular functions by loading specific macromolecular bioactive substances into the coating layers, such as VEGF 38, FGF-2 39, and IGF-1 40. Furthermore, DP has been shown to secrete transforming growth factor (TGF)-β2 *in vivo,* which counterbalances bone morphogenic proteins (BMPs), both of which belong to the transforming growth factor superfamily 41, to activate HFSCs concomitant with entry into HF regeneration 42. However, whether TGF-β2 plays a similar role *in vitro* and induces the transformation of CD34^+^ HFSCs to activated Lgr5^+^ stem cells when applied as a single-cell coating in the LbL model, requires further validation.

Therefore, the current study sought to develop a novel method to amplify stem cells and regulate their fate by constructing a nanoscale microenvironment. To this end, we first verified whether LbL coating with gelatin and alginate could be successfully applied to single HFSCs to generate nanoscale ECM. We then examined how this single cell-based LbL coating would impact the viability, proliferation, morphology, and stem cell properties of HFSCs. Next, we loaded TGF-β2 into the coating layer to investigate whether LbL coating could function as a drug carrier to regulate the fate of HFSCs. The mechanism of action of TGF-β2 on HFSCs was also investigated. Finally, we performed *in vivo* reconstitution assays to determine the hair-inductive efficiency of LbL-coated HFSCs. The results indicate that the nanoscale biomimetic microenvironment regulates HFSC quiescence and activation. Furthermore, improved HF regeneration was achieved when both activated and quiescent HFSCs were co-injected subcutaneously into nude mice.

## Methods

### Animals

Male adult (4-6 weeks old) athymic nude mice (Balb/cAJcl-nu) and newborn C57BL/6J mice were purchased from the Experimental Animal Center of Southern Medical University (Guangzhou, China). All animal studies were conducted under the approval of the Animal Care and Use Committee at the International Medical Center to reduce suffering and provide for the full protection of animal welfare.

### Cell cultures

Individual HFs was dissected from 4-6-week-old C57BL/6J mice vibrissae using micro forceps and a stereoscope. The HF tissue was digested with 0.1% dispase (Invitrogen, Carlsbad, CA, USA) for 1 h at 37 °C and shaken once every 20 min. The dermal sheaths were separated and removed under a stereoscope. The remaining HF portions were digested with 0.05% trypsin (Gibco, Gaithersburg, MD, USA) for 15-20 min. An equal volume of 10% fetal bovine serum (FBS; Gibco) in Dulbecco's modified Eagle's medium (DMEM; Gibco) was added to terminate digestion and samples were passed through a 70 µm filter (Corning, Corning, NY, USA). Following centrifugation at 300 *x g* for 5 min and washing, fluorescent activated cell sorting (FACS) was performed to isolate CD34^+^α6^+^ HFSCs. Cells were cultured in Defined Keratinocyte-serum free medium (K-SFM; Gibco) in flasks pre-coated with Geltrex (Gibco) for 1 h.

### Flow cytometry

Single-cell suspensions were prepared from HFs or cultured cells (n = 3). The cells were washed once with K-SFM and stained with fluorescently labeled antibodies for 30 min at 4 °C. Next, after washing twice with FACS buffer (2% FCS, 2 mM EDTA, PBS), cells were analyzed using an LSRFortessa (BD Biosciences, San Jose, CA, USA) or sorted using a MoFlo XDP (Beckman Coulter, Brea, CA, USA). Data were analyzed using FlowJo software version 10 (BD Biosciences). The following antibodies were used: APC-CD34 (eBioscience, San Diego, CA, USA), FITC-Lgr5 (R&D Systems, Minneapolis, MN, USA), and PE-a6 integrin (eBioscience).

### LbL nano-coating of individual HFSCs

Next, 1 × 10^6^ HFSCs were added to 15 mL centrifuge tubes, centrifuged at 300 × *g* for 5 min, and media removed. After which 2 mL of 0.1% gelatin (ThermoFisher Scientific, Waltham, MA, USA) solution was added and incubated at 4 °C for approximately 10 min with gentle shaking. After centrifugation at 300 × *g* for 5 min, the supernatant was discarded. Next, the cell pellets were washed twice with 5 mL Dulbecco's phosphate-buffered saline (DPBS; Gibco) and 2 mL of 0.1% alginate (ThermoFisher Scientific) solution was added. Then, cells were incubated for 10 min as described above. Finally, the process was repeated to coat the cells with an additional layer of gelatin. Ultimately, the process was repeated until three layers of LbL coating were applied to the HFSCs.

### Preparation of fluorescence-labeled materials

For the preparation of rhodamine B-conjugated alginate, 10 mg of rhodamine B (Invitrogen) was dissolved in 1 mL DPBS, after which 20 mg of 1-ethyl-3-(3(dimethylamino) propyl) carbodiimide (EDC; ThermoFisher Scientific) and 1.75 mg 1-hydroxy-2, 5-pyrrolidinedione (NHS; ThermoFisher Scientific) were added and incubated for 30 min. Ethylenediamine was then added and mixed overnight at room temperature (RT; i.e. 25 ± 1 °C). Following dialysis and lyophilization, rhodamine B-ethylenediamine powder was added to the alginate solution (20 mg alginate dissolved in 2 mL DPBS) and 10 mg of EDC. The solution was stirred at RT overnight and then dialyzed and lyophilized to obtain the final Rhodamine B-Alginate powder. Fluorescein isothiocyanate conjugated gelatin (Gelatin-FITC) was purchased from Invitrogen. For the preparation of FITC-conjugated TGF-β2, 30 mg of TGF-β2 was dissolved in 3 mL of 0.1 M sodium bicarbonate buffer, and 15 mg FITC (Sigma-Aldrich, St. Louis, MO, USA) was dissolved in 1 mL 0.1 M sodium bicarbonate buffer. The FITC solution was then slowly added to that of TGF-β2 under magnetic stirring. The reaction was incubated overnight at RT with continuous stirring. Finally, the FITC-conjugated TGF-β2 was obtained following dialysis and lyophilization.

### Transmission electron microscopy (TEM)

Untreated HFSCs and LbL-HFSCs were fixed with 2.5% glutaraldehyde (Solarbio, Wuhan, China) on days 1, 3, and 7 of culture and dehydrated at 4 °C for 4 h (n = 3). Ultra-thin sections were prepared and stained with uranyl acetate and lead citrate. TEM was conducted on a Tecnai-10 microscope (Philips, Amsterdam, Netherlands).

### Scanning electron microscopy (SEM)

Untreated HFSCs and LbL-HFSCs were fixed with 2.5% glutaraldehyde for 1, 3, and 7 days and dehydrated in ethanol (n = 3). The samples were subjected to gold spraying under vacuum and characterized by SEM using a JSM-6330F (JEOL, Tokyo, Japan).

### Zeta potential assessment

Untreated HFSCs and HFSCs coated with gelatin, gelatin/alginate, or (gelatin) 2/alginate were collected separately (n = 4). The zeta potential of the four sample types was determined by the instrument (Malvern Instruments, Malvern, England).

### Live/dead staining

All samples were stained on days 3 and 7 of culture using a Live/Dead Viability Kit (Invitrogen) according to manufacturer's instructions, and incubated for 15-20 min at 37 °C (n =3). Next, images were obtained using a fluorescence microscope (IX71 FL, Olympus, Tokyo, Japan).

### Cell cycle

Single-cell suspensions were prepared from cultured cells (n =3). The cells were fixed on day 7 of culture with 75% alcohol, stained with the Cell Cycle Kit (Invitrogen) according to manufacturer's instructions, and analyzed using a BD LSRFortessa. Data were analyzed using FlowJo software version 10.

### Cloning formation assay

1 × 10^3^ HFSCs/well were placed into 6-well culture plates pre-coated for 1 h with Geltrex (n =4). After 14 days of culture, the colonies were stained with hematoxylin (Solarbio). The colony number was determined from scanned images using Image-pro software version 6.0 (Media Cybernetics, Rockville, MD, USA).

### Immunofluorescence

The samples were washed once with DPBS, fixed with 4% paraformaldehyde for 15 min at RT, rinsed three times with DPBS, and then permeabilized in 0.3% Triton X-100 (Solarbio) before being blocked with 3% bovine serum albumin (BSA; Solarbio). Samples (n =3) were then stained with primary antibodies against TGF-β2 (1:200, Abcam), Ki67 (1:200, Abcam), CD34 (1:200, Abcam), cytokeratin 10 (K10; 1:100, Abcam), Lgr5 (1:100, R&D Systems), cytokeratin 15 (K15; 1:200, Abcam) and integrin a6 (a6; 1:100, Abcam) overnight at 4 °C. The next day, samples were incubated with secondary antibody (1:200, Abcam) and DAPI (1:200) for 1 h at RT. Fluorescence microscopy images were captured under a fluorescence microscope (IX71 FL, Olympus) or confocal laser scanning microscope (LSM880, Cari Zeiss, Jena, Germany).

### Drug loading and enzyme-linked immunosorbent assay (ELISA)

The procedure for drug loading was the same as for LbL nano-coating except that 100 pM TGF-β2 was added to 2 ml of 0.1% alginate for loading in the second layer. For ELISA, 50-ml samples were collected from triplicate cultures at a designated time point and then added to microplates previously coated with anti-TGF-β2 for 2 h. After adding the conjugation solution to each well for 2 h, the substrate solution was pipetted into the plate for 30 min, and then the stop solution was added. Absorbance was measured at 450 nm using a multi-label counter (n = 3).

### Knockdown experiments

FACS-isolated HFSCs loaded with TGF-β2 were seeded into 24-well plates at a density of 4 × 10^4^ cells/cm^2^ and cultured for 72 h at 37 °C. Transfection was carried out by adding 100 µL small interfering RNA (siRNA) Transfection Medium containing 10 µL Tmeff1-siRNA1 (Santa Cruz Biotechnology, Santa Cruz, CA, USA) or Tmeff1-siRNA2 (Santa Cruz Biotechnology) to the medium. The cells were incubated for an additional 96 h prior to being harvested and analyzed for gene and protein expression (n =3).

### Quantitative real-time polymerase chain reaction (qRT-PCR)

Total RNA was isolated from HFSCs using TRIzol (Invitrogen; n =3). The cDNA was synthesized from a total of 2 mg RNA using the SYBR PrimeScript RT-PCR kit, as per manufacturer's instructions. Subsequent qRT-PCR was performed using the SYBR PrimeScript RT-PCR kit on a Stratagene MX3005P QRT-PCR system (Agilent Technologies, Santa Clara, CA, USA) according to the manufacturer's protocol. The primer sequences are provided in Supplementary Table S1.

### Western blotting

Cell lysates were dissolved in a substrate-soluble buffer, electrophoresed on a 2% sodium dodecyl sulfate-polyacrylamide gel, and subjected to immunoblot analysis (n =3). After blocking with 3% BSA, the blotting membranes were exposed to the following primary antibodies: CD34 (1:1,000, Abcam), K10 (1:1,000, Abcam), Lgr5 (1:1,000, R&D Systems), Tmeff1 (1:1,000, Abcam), pSmad1/5 (1:1,000; Cell Signaling Technology, Danvers, MA, USA), ID2 (1:1,000, Abcam), and ID3 (1:1,000, R&D Systems) overnight at 4 °C. After washing, the blots were incubated with the corresponding secondary antibody (1:1,000, Abcam) for 1 h at RT and photographed with an Odyssey infrared fluorescent scanning imaging system (Li-COR Biosciences, Lincoln, NE, USA).

### Preparation of cells for *in vivo* grafting

Isolation of mouse dermal cells was performed as previously described 43. Briefly, full-thickness dorsal skin was removed from C57BL/6J mice on natal day 0 and digested with 0.1% dispase at 37 °C for 1 h. The skin specimen was then divided into epidermis and dermis using forceps. The dermis was minced and digested in 0.2% collagenase (Sigma-Aldrich, St. Louis, MO, USA) at 37 °C for 1 h. After digestion, an equal volume of 10% FBS in DMEM was added to terminate the reaction, and the samples were filtered through 70 µm strainers. Following centrifugation and washing, the mouse dermal cells were obtained.

### *In vivo* fluorescence imaging

FITC-conjugated TGF-β2 was employed for fluorescence signal tracing, and was prepared as previously described. For *in vivo* experiments, 5 × 10^5^ P1- LbL-coated HFSCs loaded with FITC-conjugated TGF-β2 or 5 × 10^5^ P1 HFSCs with FITC-conjugated TGF-β2 medium or 5 × 10^5^ P1 LbL-HFSCs, combined with 5 × 10^5^ mouse dermal cells were injected subcutaneously into the dorsal site of athymic nude mice (n =16 per group). FITC- conjugated TGF-β2 were detected using the *In vivo* FX Pro imaging system (Bruker, Madison, WI, USA) according to manufacturer's instructions, on days 1, 3, 5, and 7 post-injection. All fluorescence intensities were analyzed using the MISE software (Bruker), and are presented in terms of photon flux (photons sec^-1^, cm^-2^ steradian [SR]^-1^). On days 1, 3, 5, and 7 post-injection, the grafted regions were harvested and 10-µm thick frozen sections were obtained. Fluorescence microscopy images were captured via fluorescence microscopy (IX71 FL, Olympus).

### *In vivo* hair regeneration

For *in vivo* implantation, cells were divided into six groups, 1 × 10^6^ mouse dermal cells alone used as a control group (n =8), and 5 × 10^5^ P1-HFSCs mixed with 5 × 10^5^ mouse dermal cells, which were used in five different experimental groups (n =8 per group): HFSCs, LbL-coated HFSCs (LbL-HFSCs), HFSCs + TGF-β2 (10 pM), LbL-coated HFSCs loaded with TGF-β2 [LbL(TGF-β2)-HFSCs], and 50% LbL-HFSCs + 50% LbL (TGF-β2)-HFSCs (50% + 50%). Athymic nude mice were anesthetized with pentobarbital sodium (1.3 mg/kg body weight). Next, 1 × 10^6^ cells from each group were injected subcutaneously into the dorsal side in a total volume of 50 µL PBS using a 29-gauge needle (BD Biosciences). After 3 wk, the grafted specimens were examined under a stereomicroscope (MVX10, Olympus) and photographed. The grafted regions were harvested, fixed in 4% paraformaldehyde, and embedded in paraffin. Subsequently, 4-µm thick serial sections were stained with hematoxylin and eosin (H&E). Representative areas for each group were selected and photographed for further analysis.

### Statistical analysis

All experimental data were analyzed using SPSS 18.0 (SPSS, Inc., Chicago, IL, USA). One-way analysis of variance (ANOVA) was performed for comparison of the different groups. The results were expressed as mean ± standard deviation (SD). Each experiment was repeated a minimum of three times. Results with a *p*-value < 0.05 were considered statistically significant. All graphs were plotted using GraphPad Prism 7 (GraphPad Software, Inc., La Jolla, CA, USA).

## Results and Discussion

### Appropriately coated HFSCs and their characteristics *in vitro*

**Figure 1:**
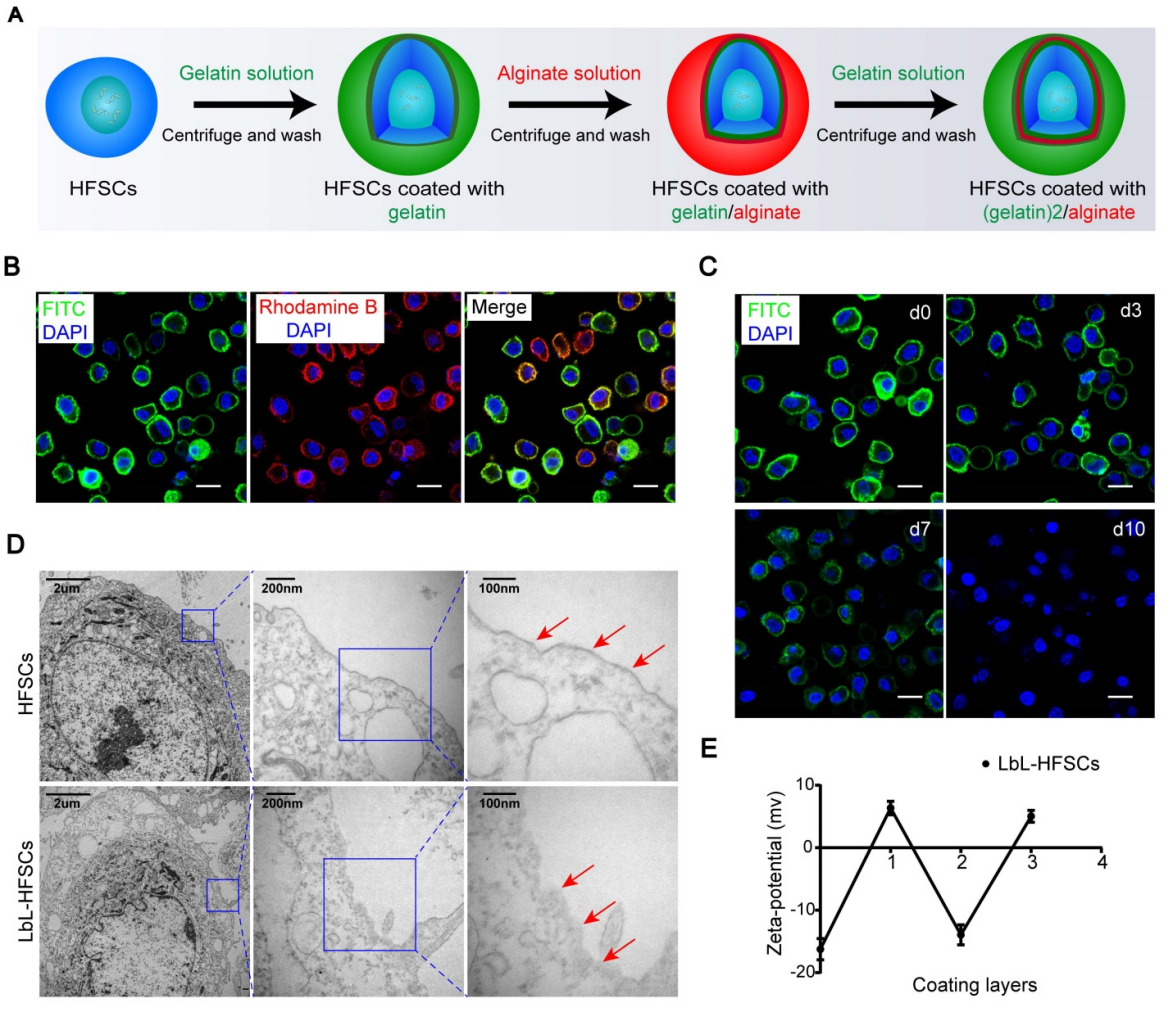
** Generation of layer-by-layer-hair follicle stem cells (LbL-HFSCs) using the LbL cell coating technique.** (A) Schematic illustration of LbL-HFSCs fabrication using gelatin (green) and alginate (red) to coat HFSCs. (B) Confocal laser scanning microscopy (CLSM) images displaying the LbL coating on HFSCs in which the cell surface was coated with gelatin-FITC (green) and alginate-rhodamine B (red). The cells were in suspension. The nuclei were stained with DAPI (blue). Scale bars: 10 µm. (C) The persistence of the biomaterial coated with LbL on the cell surface is shown in the CLSM image. The cells were in suspension. Gelatin-FITC (green); DAPI (blue); Scale bars: 10 µm. (D) Transmission Electron microscope (TEM) images show the comparison of the uncoated HFSCs and coated HFSCs. The arrows indicate coating materials on the cells' surface. (E) Corresponding changes in zeta potential according to the different layers of coating on the cell surface: (0) untreated HFSCs, (1) HFSCs coated with gelatin, (2) HFSCs coated with gelatin/alginate, and (3) HFSCs coated with (gelatin)2/alginate.

Certain ECM components have been found to play essential roles in maintaining cells in undifferentiated states 44,45. The use of native ECM proteins and polysaccharides as scaffolds creates an environment similar to that of natural *in vivo* ECM, which can exhibit desired mechanical and physical characteristics capable of mimicking *in vivo* stem cell niches 46. Gelatin is a biocompatible protein with similar properties to the native ECM 47; while alginate offers excellent biocompatibility and is widely applied in tissue engineering 48. As such, gelatin and alginate were ideal biomaterials for constructing nanoscale ECM. To construct nanoscale ECM for individual HFSCs, first, we extracted, purified, and identified HFSCs **(Figure S1; Supporting Information)**. We then coated the HFSCs with gelatin and alginate using the LbL technique. Due to the different isoelectric points (IEP), gelatin (IEP = 7-9) exhibits a positive charge while alginate (IEP = 5.4) shows a negative charge at neutral pH (7.4). A 0.1% (w/v) gelatin solution was applied to the first coating on the cell surface to provide an excess of charge followed by anionic alginate. Gelatin (polycation) and alginate (polyanion) were sequentially deposited onto the surface of the HFSCs for a total of three layers** (Figure 1A)**. To evaluate the feasibility of LbL coating on HFSCs, we employed FITC-conjugated gelatin and rhodamine B-conjugated alginate to visualize the LbL coating on the membrane surface of HFSCs. Each encapsulation showed the corresponding coating layers with either green or red fluorescence **(Figure 1B)**. Furthermore, the retention of biomaterials on the membrane surface was determined by monitoring the HFSC coating with (gelatin-FITC)-alginate-gelatin from days 0 to 10** (Figure 1C)**. The fluorescence intensity was found to gradually decrease over time, indicating that biomaterials were maintained for approximately 7 days, providing a sufficient period for their function and application.

TEM was also performed to confirm the presence of the nano-matrix coatings on LbL-HFSCs compared to that on untreated HFSCs** (Figure 1D)**. To evaluate the changes in cellular potential for the different layers of nano-coating, zeta potential assays were performed. The data showed a zigzag tendency corresponding to the coat with varied layers of oppositely charged polyelectrolytes **(Figure 1E)**. These results confirmed that LbL coating with gelatin and alginate was successfully applied to HFSCs.

### LbL coating maintained the spheroid-like morphology of HFSCs without impacting cell viability and proliferation

**Figure 2:**
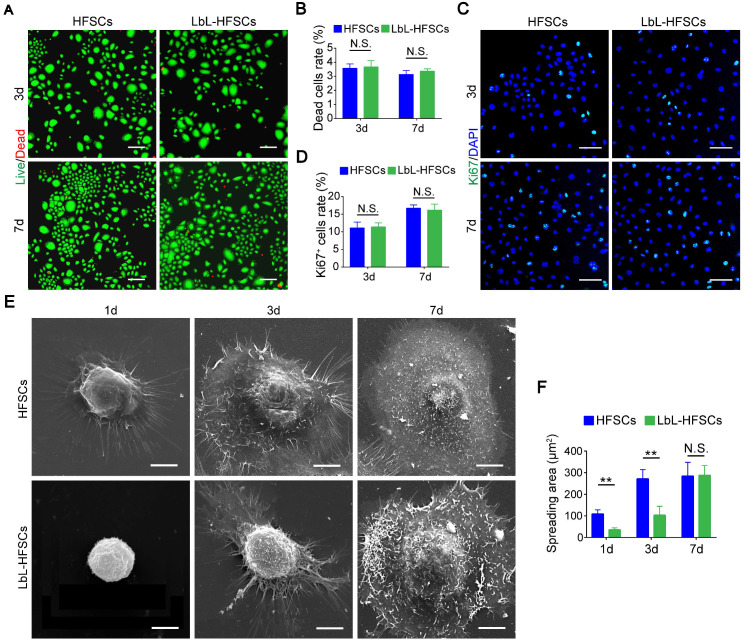
** Layer-by-layer (LbL) coating was not detrimental to cell viability and proliferation of the hair follicle stem cells (HFSCs).** (A) Live/Dead staining of both HFSCs and LbL-HFSCs on day 3 and day 7 of culturing. Live (green); Dead (red); Scale bars: 100 µm. (B) The proportion of dead cells in the HFSCs and LbL-HFSCs groups showed no significant difference (*p* > 0.05). (C) Ki67 Immunofluorescence was performed on day 3 and day 7 post-coating to evaluate cell proliferation. Ki67 (green); DAPI (blue); Scale bars: 50 µm. (D) The proportion of Ki67^+^ cells in HFSCs compared to that in the LbL-HFSCs showed no significant difference (*p* > 0.05). (E) Morphological evaluation of HFSCs and LbL-HFSCs was conducted using scanning electron microscopy (SEM) on day 1, 3, and 7 post-coating. Scale bars: 5 µm. (F) The spreading area of LbL-HFSCs was significantly lower than that of the HFSCs on day 1 and day 3 post-coating, but no significant difference was observed on day 7 (*p* > 0.05). NS, not significant; **p < 0.01.

Next, we coated individual HFSCs with gelatin and alginate using LbL self-assembly technology to construct nanoscale ECM. To elucidate how a single-cell based LbL coating with gelatin and alginate could influence the HFSCs, we examined whether the LbL coating affected viability, proliferation, or morphology of the HFSCs. Live/dead staining was performed on days 3 and 7 post-LbL coating** (Figure 2A)**. We observed only low rates of cell death in both the uncoated and LbL-coated HFSCs, without any significant differences between the groups (*p* > 0.05; **Figure 2B**). This indicates that the ECM generated by LbL coating was not detrimental to HFSC cell viability. Immunostaining for Ki67 was then performed to assess the proportion of proliferating cells in both groups on days 3 and 7 post-coating **(Figure 2C)**. As shown in **Figure 2D**, no significant differences in the proliferation of uncoated and LbL-coated HFSCs were detected (*p* > 0.05). For evaluation of cell morphology, we performed SEM analysis on the uncoated and LbL-coated HFSCs on days 1, 3, and 7 post-coating** (Figure 2E)**. LbL-coated HFSCs exhibited spheroid-like morphology at early timepoints post-coating, while uncoated HFSCs displayed a broadly stretched morphology. However, no difference was observed between the two groups on day 7 post-coating **(Figure 2F)**, suggesting that the LbL-coated HFSCs may exhibit more physiologically-relevant morphological 3D structures at early timepoints post coating.

### LbL coating enables the maintenance of stem cell properties in HFSCs

**Figure 3:**
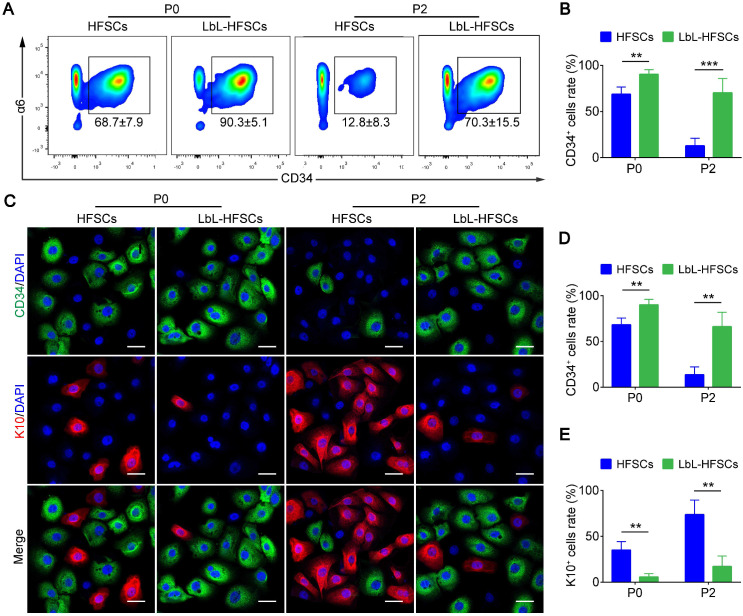
** Layer-by-layer (LbL) coating maintained hair follicle stem cells (HFSCs) properties.** (A-B) Flow cytometry was performed on day 7 post-coating, demonstrating that the ratio of CD34^+^ cells in LbL-HFSCs was significantly higher than that in the HFSCs both at P0 and P2. (C-E) Immunofluorescence staining was performed on day 7 post-coating at P0 and P2, and the ratio of CD34^+^ cells was significantly higher in LbL-HFSCs compared to that in HFSCs, while the ratio of K10 was significantly lower. CD34 (green); K10 (red); DAPI (blue); Scale bars: 20 µm; **p < 0.01; ***p < 0.001.

Further, for the *in vitro* culture of stem cells (especially HFSCs); we were most concerned with LbL coating affecting the stem cell properties. To evaluate this, for every 10k HFSCs from FACS-isolated CD34^+^ primary HFSCs were cultured with or without LbL treatment, and LbL-treated HFSCs were coated again during each passage. Flow cytometry and immunofluorescence staining were performed to determine the ratio of CD34^+^ stem cells at P0 and P2 after 7 days of culture **(Figure 3A, 3C)**. As shown in** Figure 3B and 3D**, the ratio of CD34^+^ cells in the LbL-coated HFSCs was significantly higher than that in the uncoated HFSCs with the ratio of CD34^+^ cells > 90% at P0 and > 70% at P2 in LbL-HFSCs; while the ratio of CD34^+^ cells was < 70% at P0 and < 15% at P2 in the uncoated HFSCs, the uncoated HFSCs could not be further passaged. We also examined the differentiation direction of the uncoated HFSCs via immunofluorescent staining of K10, which is an epidermal cell marker. Results confirmed differentiation of HFSCs into epidermal cells** (Figure 3C, 3E)**. The results of qRT-PCR and western blot analysis further validated the above conclusions. Expression of CD34 mRNA and protein in the LbL-coated HFSCs was significantly higher than that in the uncoated HFSCs, while the expression of K10 mRNA and protein was significantly lower (*p* < 0.05; **Figure S2**).

Overall, these results confirmed that LbL coating did not affect the viability or proliferation of HFSCs, and helped to better maintain their stem cell properties *in vitro*. Furthermore, the SEM analysis results showed that LbL-coated HFSCs exhibited *in vivo*-like 3D structures early post-coating, suggesting that the LbL modification may have provided mechanical support for HFSCs, placing them in a spherical and sparsely distributed state similar to that of the native stem cell niche. Alternatively, the LbL self-assembly technique differs from that of ECM-coated culture surfaces or gels in that cell surface modifications can be used to physically and chemically remodel niches at the individual-cell level. Taken together these results suggest that LbL-coating with gelatin and alginate may be suitable for constructing nanoscale ECM for HFSCs, thereby providing a novel strategy for *in vitro* culturing to amplify the truly multipotent HFSCs and facilitate the development of a new approach for regenerative medicine.

### LbL-coating as a sustained release drug carrier by loading TGF-β2 into the nano-coating layers

**Figure 4:**
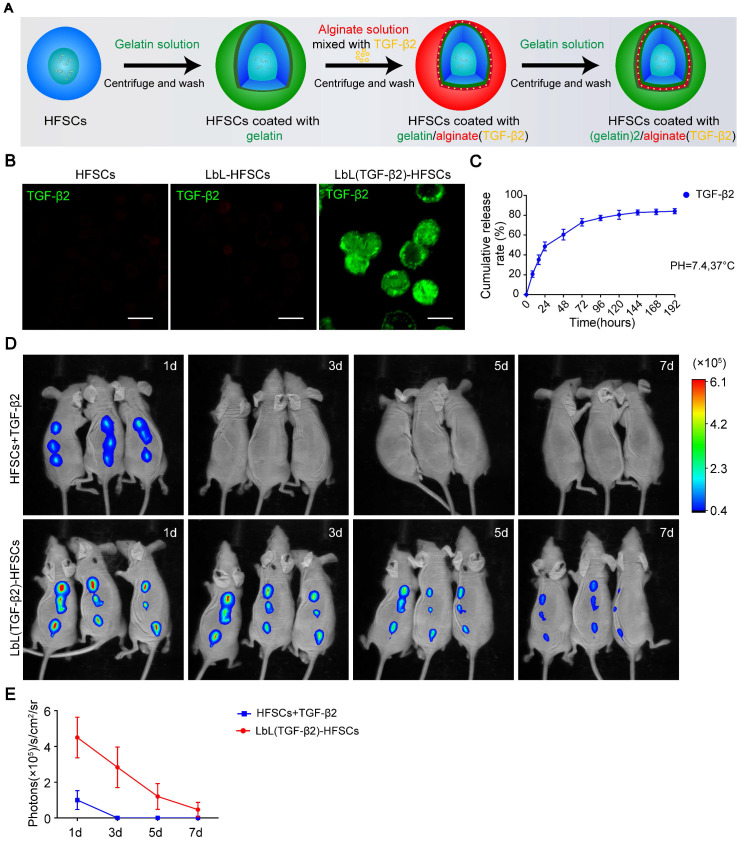
** Loading of transforming growth factor (TGF)-β2 into hair follicle stem cells (HFSCs) using layer-by-layer (LbL) techniques.** (A) Schematic diagram of LbL(TGF-β2)-HFSC preparation by coating HFSC successively with gelatin (green), alginate [red; mixed with TGF-β2 (yellow)] and gelatin (green). (B) Immunofluorescence staining of anti-TGF-β2 verified the presence of TGF-β2 only around the cell surface of LbL(TGF-β2)-HFSCs. TGF-β2 (green); Scale bars: 50 µm. (C) At various time points post-coating, the cumulative release profile of TGF-β2 was determined by ELISA. (D) *In vivo* fluorescence imaging following subcutaneous injection of HFSCs + TGF-β2 or LbL(TGF-β2)-HFSCs with FITC-conjugated TGF-β2. (E) Quantitative analysis of the FITC signal intensity expressed as photons/s/cm^2^/sr.

The activation of HFSCs is essential for HF regeneration. Determining whether the LbL coating structure could be used as a drug carrier to regulate the fate of HFSCs through the loading of bioactive molecules was critical for evaluating stem cell microenvironment construction as this would determine the extensiveness of potential applications for LbL nano-coating. Therefore, we loaded the cytokine TGF-β2 into alginate, coated the HFSCs, and coated the cells with gelatin to generate LbL(TGF-β2)-HFSCs **(Figure 4A)**. To characterize the presence of TGF-β2 on the cell surface, we incubated both the LbL-coated and uncoated HFSCs with an anti-TGF-β2 antibody. Only the LbL(TGF-β2)-HFSCs showed positive results **(Figure 4B)**. The release profile is shown in **Figure 4C**. The LbL(TGF-β2)-HFSCs exhibited a sustained and time-dependent release profile that lasted for approximately 7 days in a physiological environment (pH = 7.4, T = 37 °C), providing sufficient time to regulate the cell function *in vitro*. Next, to determine whether LbL(TGF-β2)-HFSCs could be used as a drug carrier to regulate HFSCs function *in vivo*, athymic nude mice were subcutaneously injected with HFSCs + TGF-β2 or LbL(TGF-β2)-HFSCs, of which TGF-β2 was FITC-conjugated. The mice were imaged using an IVIS fluorescence imaging system from days 0 to 7** (Figure 4D)**. The intensity of fluorescence gradually diminished and decreased to a nearly undetectable level by day 7 in LbL(TGF-β2)-HFSCs, while a weak fluorescence intensity was detected only on the first day in HFSCs + TGF-β2 **(Figure 4E)**. Skin sections obtained near the injection site further confirmed that the LbL coating maintained TGF-β2 for approximately 7 days **(Figure S3)**, providing sufficient time for regulating HFSC fate *in vivo*.

### Determining the optimal drug adding and loading concentration

**Figure 5:**
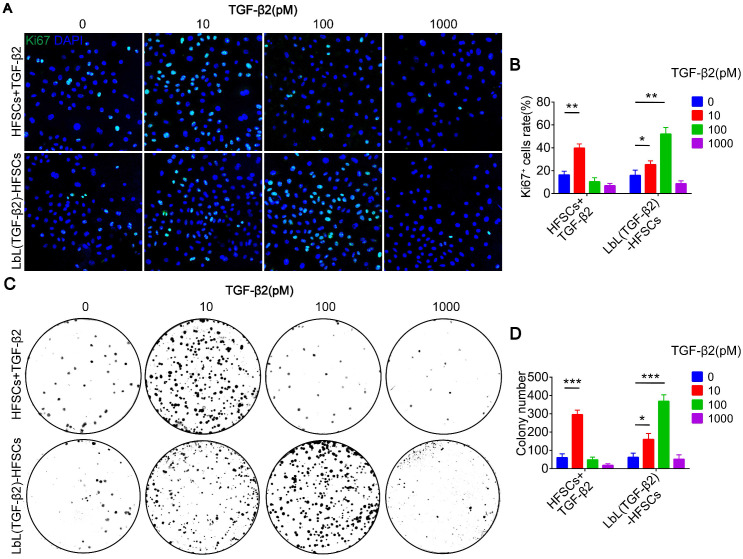
** Different drug adding and loading concentration on the proliferation of hair follicle stem cells (HFSCs).** (A) Ki67 immunofluorescence staining was performed on day 7 to evaluate the proliferation of HFSCs + TGF-β2 and LbL(TGF-β2)-HFSCs. Ki67 (green); DAPI (blue); Scale bars: 50 µm. (B) The proportion of Ki67^+^ cells was significantly higher when TGF-β2 was added at 10 pM or loaded at 100 pM. (C-D) Colony formation assays further confirmed that cell proliferation was significantly higher when the HFSCs were added with 10 pM or loaded with 100 pM TGF-β2. *p < 0.05; **p < 0.01; ***p < 0.001.

After successfully loading of TGF-β2, we then determine the optimal drug adding and loading concentrations. Immunostaining of Ki67 and colony formation assays were performed to detect the proliferation of cells at different concentrations **(Figure 5A, 5C)**. Additionally, the most suitable drug adding and loading concentrations were determined at 10 pM and 100 pM, respectively** (Figure 5B, 5D)**. These results confirmed the successful loading of TGF-β2 and demonstrated its readiness for use in the subsequent follow-up experiments aimed at regulating the fate of HFSCs both *in vitro* and* in vivo*.

### Loading of TGF-β2 activates HFSCs and converts them into Lgr5^+^ cells

**Figure 6:**
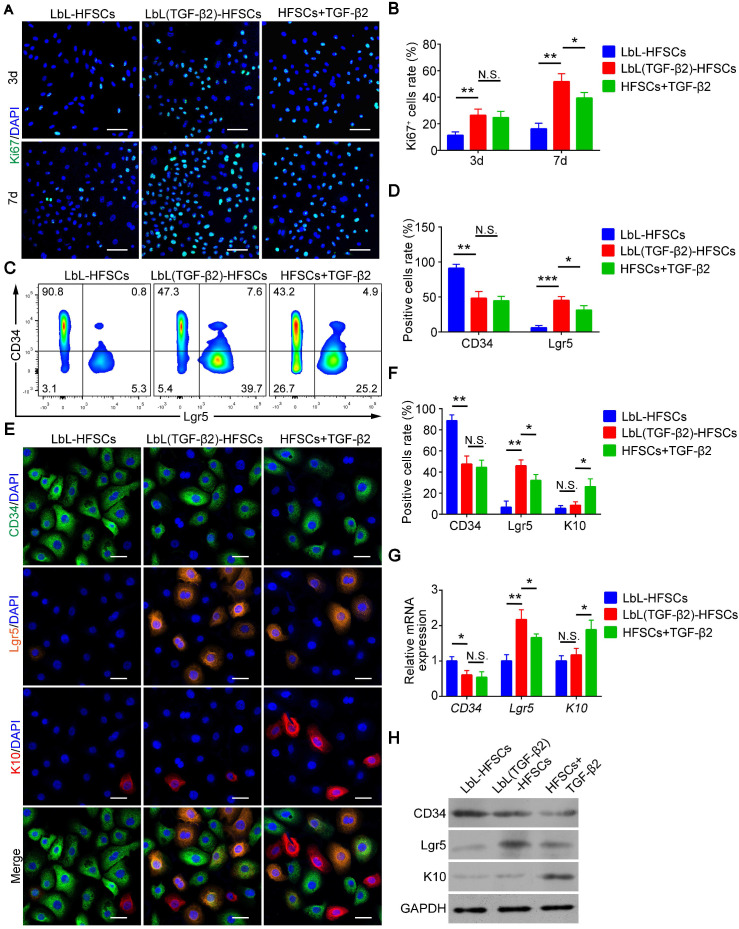
** Transforming growth factor (TGF)-β2 loading activated hair follicle stem cells (HFSCs) and converted them into Lgr5^+^ cells.** (A) Ki67 immunofluorescence staining was performed on day 3 and day 7 post-coating to evaluate cell proliferation. Ki67 (green); DAPI (blue); Scale bars: 50 µm. (B) LbL(TGF-β2)-HFSCs had higher proportions of Ki67^+^ cells than LbL-HFSCs on day 3 and day 7, LbL(TGF-β2)-HFSCs also had higher proportions of Ki67^+^ cells than HFSCs + TGF-β2 on day 7. (C) After 7 days of culture, flow cytometry was used to measure the ratio of CD34^+^ cells and Lgr5^+^ cells. (D) Flow cytometry analysis showed that LbL(TGF-β2)-HFSCs downregulated the ratio of CD34^+^ cells and upregulated the ratio of Lgr5^+^ cells. LbL(TGF-β2)-HFSCs also had higher ratio of Lgr5^+^ cells than HFSCs + TGF-β2. (E) Immunofluorescence staining was performed to detect the ratio of CD34^+^, Lgr5^+^, and K10^+^ cells on day 7. CD34 (green); Lgr5; (orange); K10 (red); DAPI (blue); Scale bars: 20 µm. (F) Compare to LbL-HFSCs, LbL(TGF-β2)-HFSCs downregulated the proportion of CD34^+^ cells and upregulated the proportion of Lgr5^+^ cells, while the proportion of K10^+^ cells showed no difference (*p* > 0.05). HFSCs + TGF-β2 had lower proportion of Lgr5^+^ cells and higher proportion of K10^+^ cells than LbL(TGF-β2)-HFSCs. (G) The qRT-PCR analysis of *CD34*, *Lgr5*, and *K10* mRNA expression. The qRT-PCR results are shown as the fold-change relative to their expression in LbL-HFSCs. (H) Western blot analysis of CD34, Lgr5, and K10 protein expression. NS, not significant; *p < 0.05; **p < 0.01; ***p < 0.001.

In addition to the development of nanoscale ECM, we were also committed to regulating the fate of stem cells and to converting CD34^+^ HFSCs to activated Lgr5^+^ cells *in vitro*. To determine whether TGF-β2 effectively activates HFSCs *in vitro*, cells were immunostained for Ki67 to evaluate cell proliferation **(Figure 6A)**. The results showed that the proliferative activity of LbL(TGF-β2)-HFSCs was significantly higher than that of the LbL-HFSCs after day 3 or day 7 of culturing. LbL(TGF-β2)-HFSCs also had higher proliferative activity compared to HFSCs + TGF-β2 at day 7 of culturing. A proliferation ratio of > 50% was achieved on day 7** (Figure 6B)**. The results were further verified by cell cycle analysis. The S-phase cell rates for the LbL(TGF-β2)-HFSCs was significantly higher than for LbL-HFSCs** (Figure S4)**. Together these results confirm that TGF-β2 loading could promote HFSC activation, inducing higher proliferative capacity compared to direct addition of TGF-β2 to the culture medium. Live/dead staining was also performed on days 3 and 7 post-TGF-β2 loading. We observed a low level of dead cells in both groups, with no significant differences detected between groups (*p* > 0.05; **Figure S5**), confirming that TGF-β2 loading did not affect cell viability.

Moreover, Hoeck et al. confirmed that HFs is unable to enter anagen when Lgr5^+^ cells are ablated 34. This indicates that Lgr5^+^ cells are essential for HF regeneration. However, *in vitro* studies regarding the conversion of CD34^+^ HFSCs to activated Lgr5^+^ cells are still lacking. We, therefore, sought to obtain Lgr5^+^ cells *in vitro*. To further determine whether TGF-β2 could regulate HFSCs and convert them from CD34^+^ cells to Lgr5^+^ cells, immunofluorescence and flow cytometry were performed **(Figure 6C, 6E)**. The results showed that TGF-β2 loading downregulated the proportion of CD34^+^ cells and upregulated the proportion of Lgr5^+^ cells. Although the LbL(TGF-β2)-HFSCs and HFSCs + TGF-β2 did not exhibit significant differences in the proportion of CD34^+^ cells, LbL(TGF-β2)-HFSCs did have a higher proportion of Lgr5^+^ cells **(Figure 6D, 6F)**. In addition, the proportion of K10^+^ cells in HFSCs + TGF-β2 was higher than in LbL(TGF-β2)-HFSCs **(Figure 6E-F)**. Further, the results of qRT-PCR and western blot analysis confirmed the above findings **(Figure 6G-H)**. Hence, TGF-β2 loading may serve to significantly down-regulate the expression level of CD34 mRNA and protein, while up-regulating the expression of Lgr5 mRNA and protein. In addition, Lgr5 mRNA and protein expression levels of LbL(TGF-β2)-HFSCs were higher than in HFSCs + TGF-β2, while K10 mRNA and protein expression levels were lower. These findings verify that TGF-β2 loading may regulate the fate of HFSCs more efficiently than that of HFSCs + TGF-β2, while inducing the transformation of CD34^+^ cells to Lgr5^+^ activated cells rather than to epidermal cells.

### TGF-β2 target gene *Tmeff1* inducing in HFSC activation

**Figure 7:**
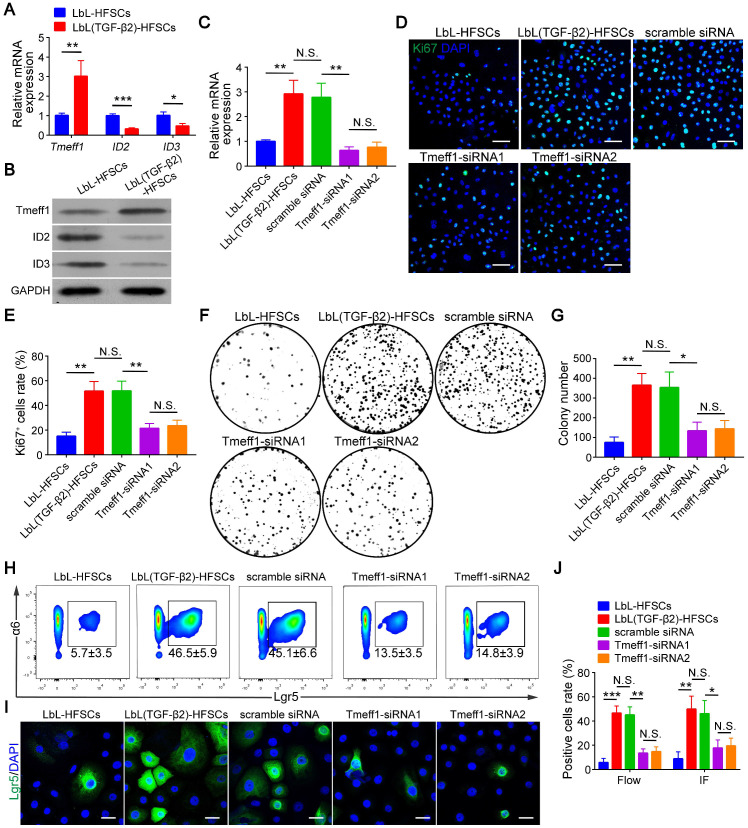
** Transforming growth factor (TGF)-β2 target gene Tmeff1 activated hair follicle stem cells (HFSCs).** (A) qRT-PCR analysis of *Tmeff1*, *ID2*, and *ID3* mRNA expression. Results are shown as the fold-change relative to the expression in LbL-HFSCs. (B) Western blot analysis of Tmeff1, ID2, and ID3 protein expression. (C) LbL(TGF-β2)-HFSCs were transfected with scramble siRNA or two different siRNAs targeting *Tmeff1* (*Tmeff1*-SiRNA1 and *Tmeff1*-SiRNA2). qRT-PCR analysis of *Tmeff1* mRNA expression. Results are shown as the fold-change relative to the expression in LbL-HFSCs. (D) Ki67 immunofluorescence staining was performed on day 7 post-coating to evaluate cell proliferation. Ki67 (green); DAPI (blue); Scale bars: 50 µm. (E) Knockdown of *Tmeff1* lowered the proportion of KI67^+^ cells. (F-G) Colony formation assays further confirmed that the cell proliferation activity was lowered by the knockdown of *Tmeff1*. (H-I) After 7 days of culturing, flow cytometry and immunofluorescence staining were used to evaluate the ratio of Lgr5^+^ cells. Lgr5 (green); DAPI (blue); Scale bars: 20 µm. (J) Knockdown of *Tmeff1* downregulated the proportion of Lgr5^+^ cells. NS, not significant; *p < 0.05; **p < 0.01; ***p < 0.001.

Next, we evaluated the molecular mechanism of TGF-β2 activated-HFSCs. BMP signaling is the critical pathway for maintaining the quiescent state of HFSCs, and its BMP/Smad1/5 encoded gene IDs serve as markers for the inhibition of HFSC activation 42. Previous studies have shown that TGF-β2 inhibits BMP signaling through the TGF/Smad2/3 pathway target gene *Tmeff1* and induces the activation of HFSCs 49. The qRT-PCR and western blot results of our study demonstrated that Tmeff1 was upregulated in LbL(TGF-β2)-HFSCs; while ID2 and ID3 encoded by BMP were downregulated **(Figure 7A-B)**. Therefore, we hypothesized that TGF-β2 activates HFSCs by blocking BMP signaling and down-regulating the marker gene IDs through the target gene, *Tmeff1.* To test this hypothesis, we first determined if TGF-β2 could activate HFSCs through Tmeff1. To this end, we added *Tmeff1*-siRNA1 and *Tmeff1*-siRNA2 to the LbL(TGF-β2)-HFSCs. Subsequent qRT-PCR results revealed that the two different siRNAs successfully downregulated *Tmeff1* mRNA expression post-transfection, as compared to untransfected HFSCs (LbL(TGF-β2)-HFSCs) or mock transfected HFSCs (scramble siRNA). No significant difference was observed in knockdown efficiency between *Tmeff1*-siRNA1 and *Tmeff1*-siRNA2 **(Figure 7C)**. Ki67 immunofluorescence staining results demonstrated that the knockdown of *Tmeff1* decreased the HFSC proliferation with the ratio of Ki67^+^ cells in *Tmeff1*-siRNA HFSCs being significantly reduced (*p* < 0.05; **Figure 7D-E**). This conclusion was further confirmed by colony formation assays **(Figure 7F-G)**. In addition, flow cytometry and immunofluorescence analysis further confirmed that the TGF-β2 target gene, *Tmeff1* could induce the conversion of HFSCs to activated Lgr5^+^ cells, with the proportion of Lgr5^+^ cells in *Tmeff1*-siRNA HFSCs found to be significantly downregulated** (Figure 7H-J)**. These results indicate that TGF-β2 activates HFSCs through its target gene, *Tmeff1*.

### Tmeff1 counterbalances BMP signaling and downregulates its target gene IDs

**Figure 8:**
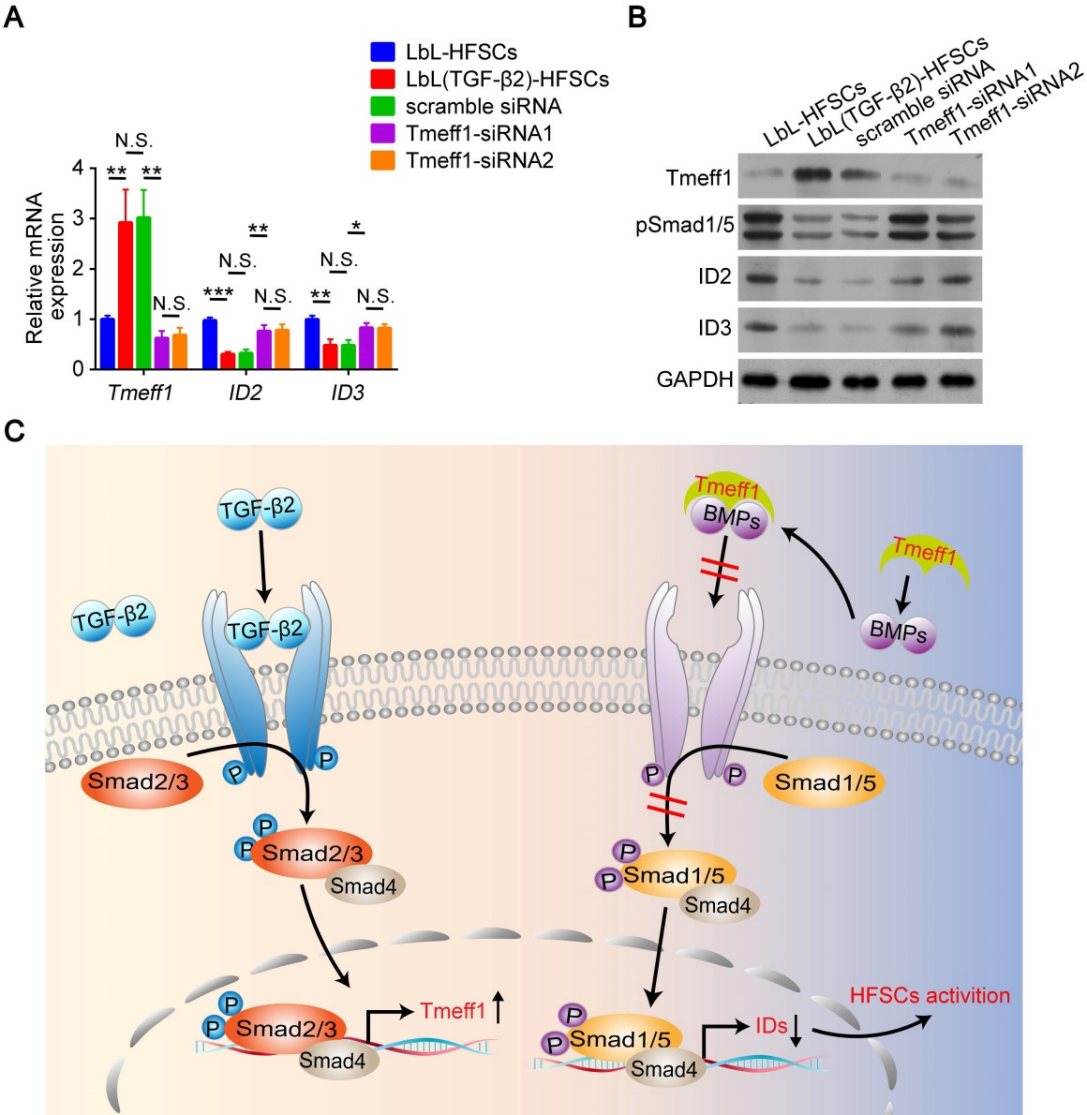
** Tmeff1 dampened BMP signaling to activate hair follicle stem cells (HFSCs).** (A) qRT-PCR analysis of *Tmeff1*, *ID2*, and *ID3* mRNA expression. Results are shown as the fold-change relative to the expression in LbL-HFSCs. (B) Western blot analysis of Tmeff1, pSmad1/5, ID2, and ID3 protein expression. (C) TGF-β2/Smad2/3 target gene *Tmeff1* counterbalanced BMP and downregulated the IDs, thereby inducing the activation of HFSCs. NS, not significant; *p < 0.05; **p < 0.01; ***p < 0.001.

This was determined via qRT-PCR, which found that *Tmeff1* was upregulated in LbL(TGF-β2)-HFSCs, while *ID2* and *ID3* encoded by BMP were downregulated. However, following *Tmeff1* knockdown, *ID2* and *ID3* gene expressions were partially restored **(Figure 8A)**. Western blot analysis further confirmed the above findings. Furthermore, Tmeff1 was upregulated in LbL(TGF-β2)-HFSCs, while BMP signaling protein pSmad1/5 and its encoded proteins ID2 and ID3 were downregulated. However, following knockdown, the expression of Tmeff1 was downregulated and the expression of pSmad1/5, ID2, and ID3 was partially restored **(Figure 8B)**. Therefore, our study demonstrated that the TGF-β2/Smad2/3 target gene, *Tmeff1,* inhibited BMP signaling and downregulated its target genes *ID2* and *ID3*, leading to the activation of HFSCs **(Figure 8C)**.

### Co-transplantation of CD34^+^ cells with Lgr5^+^ cells promotes HF inductivity

**Figure 9:**
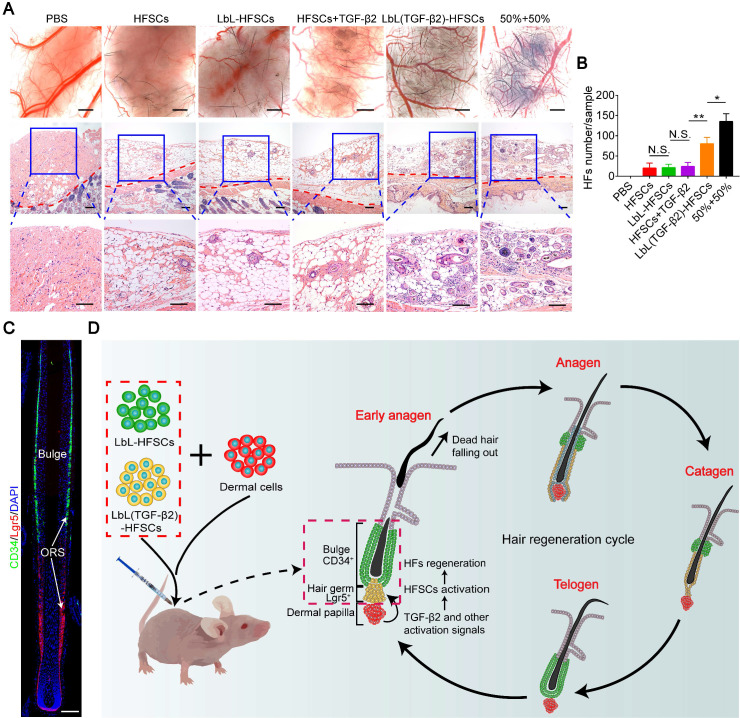
***In vivo* study of hair follicle stem cells (HFSCs) to induce new hair follicles (HFs).** (A) Stereoscopic images and hematoxylin and eosin (H&E) staining of recipient sites after 3 wk post-transplant (the red dotted line divides the injection area and mouse skin). Six groups were each co-transplanted with neonatal mouse dermal cells. Rare HF regeneration was observed in HFSCs, LbL-HFSCs and HFSCs + TGF-β2, while abundant *de novo* HFs were induced by 50% LbL-HFSCs+50% LbL(TGF-β2)-HFSCs (50% + 50%), and LbL(TGF-β2)-HFSCs. Scale bars: Stereoscopic images 500 µm and H&E images 200 µm. (B) The 50% + 50% group was the most effective population to induce HF regeneration. (C) Immunofluorescence staining was used to observe the distribution of HFSCs in *de novo* HFs. CD34^+^ stem cells were located in the bulge region, and Lgr5^+^ stem cells were situated at the lower outer root sheath (ORS). CD34 (green); Lgr5 (red); DAPI (blue); Scale bars: 50 µm (D) Schematic illustration of *in vivo* co-transplantation. LbL-HFSCs (green) and LbL(TGF-β2)-HFSCs (yellow), respectively constructed the quiescent CD34^+^ stem cells located in the bulge region (green) and the activated Lgr5^+^ stem cells in the hair germ (yellow). This mimics the two states of stem cells in hair follicle regeneration *in vivo*. NS, not significant; *p < 0.05; **p < 0.01.

A critical goal for establishing relevant stem cell microenvironments is tissue regeneration. The HFSC *in vitro* study was primarily focused on HF regeneration. To evaluate HF inductivity, we grouped control-PBS, HFSCs, LbL-HFSCs, HFSCs + TGF-β2, LbL(TGF-β2)-HFSCs, and 50% LbL-HFSCs + 50% LbL(TGF-β2)-HFSCs (50% + 50%) and combined them with neonatal mouse dermal cells, which were then subcutaneously injected into the dorsal of nude mice. The results of stereoscopic imaging and H&E staining showed that the 50% + 50% group had the most active cell population for the regeneration of HFs **(Figure 9A)**. Besides, LbL(TGF-β2)-HFSCs initiated HF growth with approximately 60% efficiency of the 50% + 50% group. Comparatively, HFSCs, LbL-HFSCs and HFSCs + TGF-β2 had lower HF inductivity, and the control group exhibited no hair growth **(Figure 9B)**. These findings verify that TGF-β2 loading induces HF regeneration of HFSCs more efficiently than that of HFSCs + TGF-β2, and the 50% + 50% group further promotes HF inductivity. In addition, immunofluorescence staining for HFSC markers was performed on the injection sites to observe the morphology and HFSC distribution of *de novo* HFs. The results showed that the *de novo* HFs had normal morphology and were in the anagen phase, CD34^+^ stem cells were observed in the bulge region, and that Lgr5^+^ stem cells were located in the lower outer root sheath (ORS). Consistent with previous reports, during the anagen phase, the Lgr5^+^ cells were located in the lower ORS and did not overlap with the CD34^+^ cells population. Additionally, Lgr5^+^ cells proliferation and migration to contributing to almost all structures of the anagen HF 37. Furthermore, to detect the safety of the prolonging release of TGF-β2* in vivo*, we stained the grafted regions of mice at 3 wk or 6 wk after LbL(TGF-β2)-HFSC transplantation with H&E and observed no tumorigenesis or fibrosis at the transplant sites **(Figure S6)**.

In summary, our previous studies confirmed that LbL-HFSCs primarily represent CD34^+^ cells, while LbL(TGF-β2)-HFSCs largely represent highly proliferating Lgr5^+^ cells *in vitro*. Furthermore, a previous study demonstrated that flow-sorted Lgr5^+^ HFSCs exhibit higher HF inductivity compared to other epidermal cells 37. This is consistent with our current experimental results which demonstrate that *in vivo* transplantation of LbL(TGF-β2)-HFSCs allowed for improved HF regeneration as compared to LbL-HFSCs. In addition, our results indicated that co-transplantation of LbL-HFSCs and LbL(TGF-β2)-HFSCs effectively reconstituted the largest number of HFs. We hypothesize that this was the result of our approach more accurately mimicking the actual biological states of HFSCs during HF regeneration *in vivo*, which includes partial activation during the early anagen phase, while other cells remain quiescent. We also established a nanoscale microenvironment for individual HFSCs by separately constructing LbL-HFSCs and LbL (TGF-β2)-HFSC *in vitro* using LbL self-assembly technology. Via* in vivo* co-transplantation, LbL-HFSCs accounted for the quiescent CD34^+^ stem cells located in the bulge, while LbL(TGF-β2)-HFSCs served as the activated Lgr5^+^ stem cells in the hair germ. This model mimics the two states of stem cells that occur during HF regeneration **(Figure 9C)** and avoids the obstacles inherent to tissue regeneration caused by stem cell activation disorder, or stem cell depletion due to excessive activation.

## Conclusions

In this study, we developed a novel method for constructing nanoscale biomimetic microenvironments for individual HFSCs. LbL self-assembly technology effectively provided means to generate nanoscale biomimetic ECMs for HFSCs, while stably expanding and maintaining their stem cell characteristics. TGF-β2, as a molecular signal, was loaded into the coating layer to regulate the fate of HFSCs. LbL coating and TGF-β2 loading led to the reconstruction of the quiescent and activated states of HFSCs, respectively, during HF regeneration. When combined with the transplantation of CD34^+^ and Lgr5^+^ HFSCs, the approach promoted HF inductivity. Hence, this method not only provides a novel approach for stem cell microenvironment construction and HF regeneration, but also confers a potential therapeutic strategy for treating hair loss diseases.

## Supplementary Material

Supplementary figures and tables.

## Abbreviations

DPCs: dermal papilla cells
ECM: extracellular matrix
HFs: hair follicles
HFSCs: hair follicle stem cells
LbL: layer-by-layer
Lgr5^+^: leucine-rich repeat-containing G-protein-coupled receptor 5^+^
TGF-β2: transforming growth factor β2
