# Supplementary material for: Nanoscale microenvironment engineering based on layer-by-layer self-assembly to regulate hair follicle stem cell fate for regenerative medicine
Source: Theranostics. 2020 Sep 22;10(25):11673–89. doi: 10.7150/thno.48723 (PMC7545990; doi:10.7150/thno.48723)
Supplement: Supplementary file 1 — Supplementary figures and tables. [file thnov10p11673s1.pdf]

Supplementary Material

I. Supplementary Tables

Supplementary Table S1. Primers used for quantitative real-time polymerase chain reaction

Gene name	Sequences (5' to 3')	
	Forward primer	Reverse primer
CD34	GAGCCACCAGAGCTATTCCC	CCAAGATGGCCAGCAGAACT
K10	AACTGACAATGCCAACGTGC	TAGGTAGGCCAGCTCTTCGT
Lgr5	CCAGTGTTGTGCATTTGGGG	CTAGCAAGGGGATTGTGGCA
Tmeff1	CAGTATCGGCCAG-ATGTGAAAGAT	TGCCTCCGCCCTCTATTGTT
ID2	ATCCCCCAGAACAAGAAGGT	TGTCCAGGTCTCTGGTGATG
ID3	GCATGGATGAGCTTCGATCT	ACCAGCGTGTGCTAGCTCTT

II. Supplementary Figures and Legends

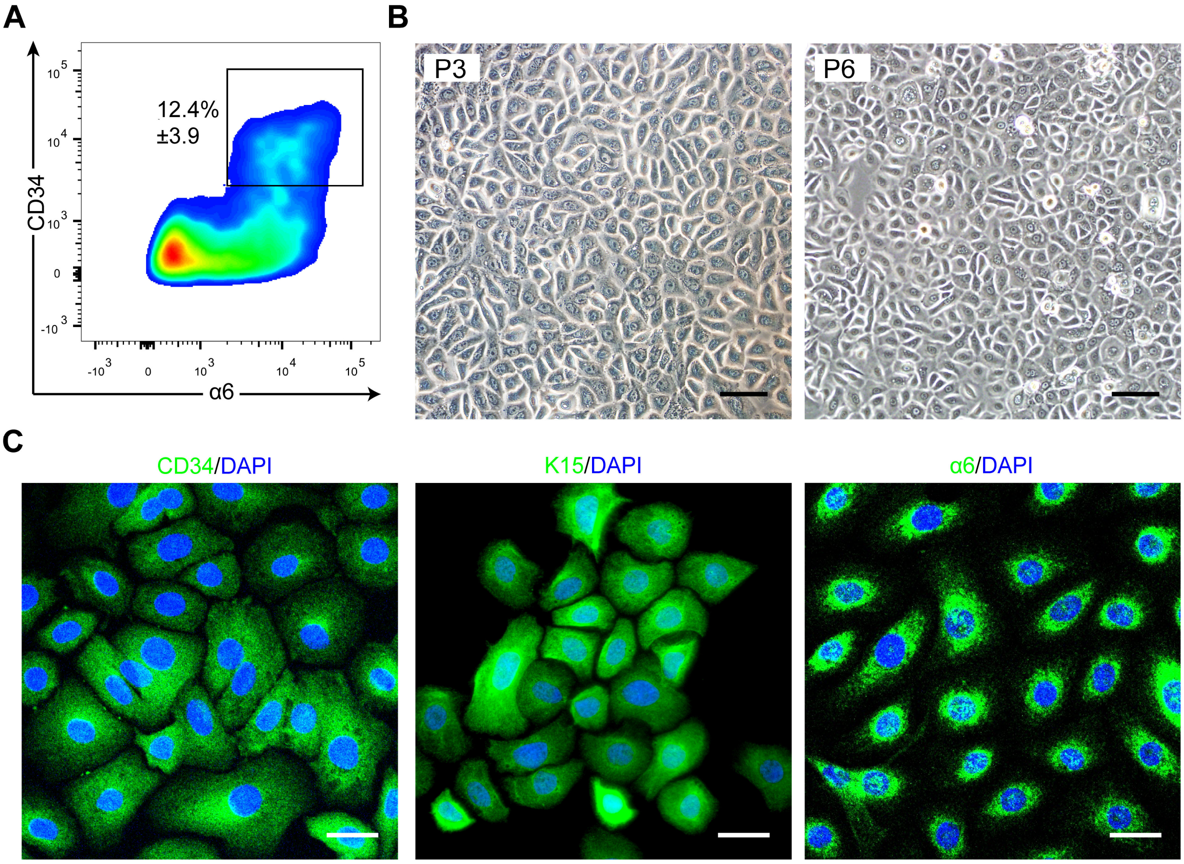

Figure S1. Purification, culturing, and identification of hair follicle stem cells (HFSCs). (A) FACS of freshly isolated vibrissae HFSCs demonstrating the ratio of CD34^{+}a6^{+} HFSCs being 12.4 ± 3.9% (± SD). (B) FACS-purified HFSCs were cultured using layer-by-layer (LbL) coating and examined by microscopy at passage 3 (p3) and passage 6 (p6). Scale bars: 100 µm. (C) LbL-coated HFSCs cultured for 7 days at P1 were immunofluorescent stained for stem cell markers CD34, cytokeratin 15 (K15), and integrin a6 (a6). All three proteins were positively expressed. CD34, K15, and a6 (green); DAPI (blue); Scale bars: 20 µm.

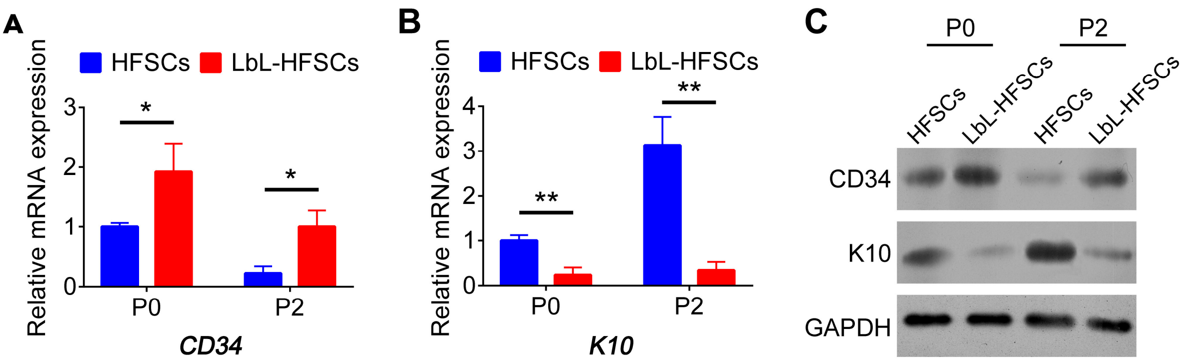

Figure S2. Stem cell properties of layer-by-layer (LbL)-coated hair follicle stem cells (HFSCs). (A–B) Quantitative reverse transcription-polymerase chain reaction (qRT-PCR) analysis of *CD34* and *K10* mRNA expression. The results show that in P0 or P2, the expression of *CD34* in LbL-HFSCs was significantly higher than HFSCs, while the expression of *K10* was significantly lower. (B) Western blot analysis of CD34 and K10 protein expression. LbL-HFSCs exhibited higher expression of CD34 and lower expression of K10 in P0 or P2. *p < 0.05; **p < 0.01.

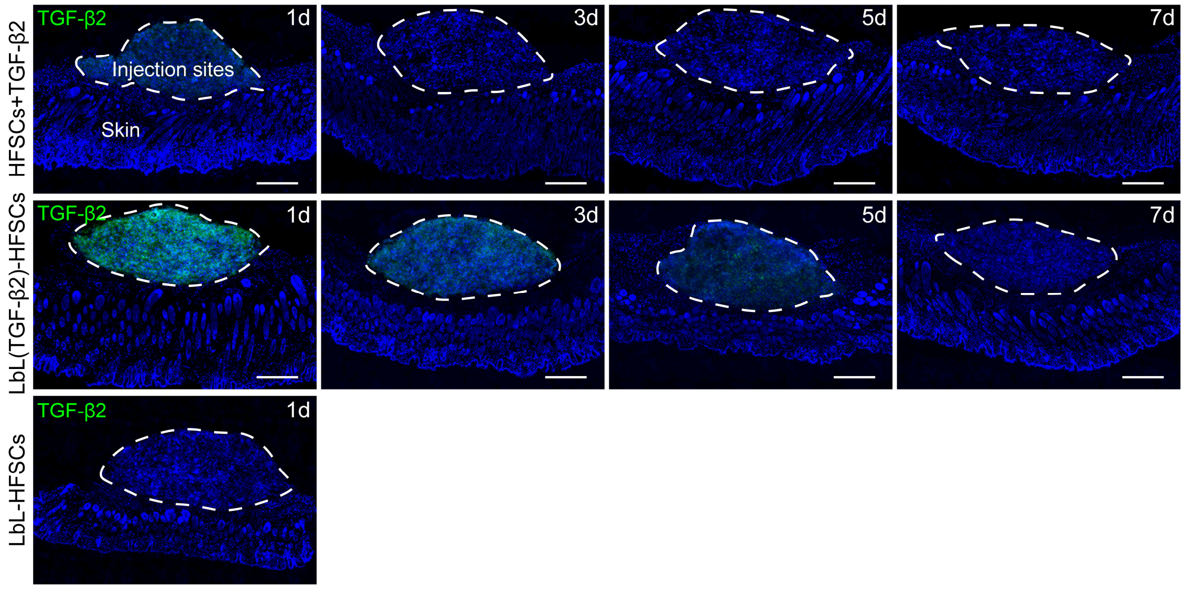

Figure S3. Layer-by-layer (LbL) coating maintained transforming growth factor (TGF)-β2 *in vivo*. *In vivo* skin sections around the injection sites showed LbL coating maintained TGF-β2 for about 7 days, while in HFSCs + TGF-β2 a weak fluorescence

intensity was detected only on the first day. Additionally, LbL-HFSCs did not exhibit green fluorescence, indicating that the fluorescence was an accurate readout of TGF-β2 activity. TGF-β2 (green); Scale bars: 500 μm.

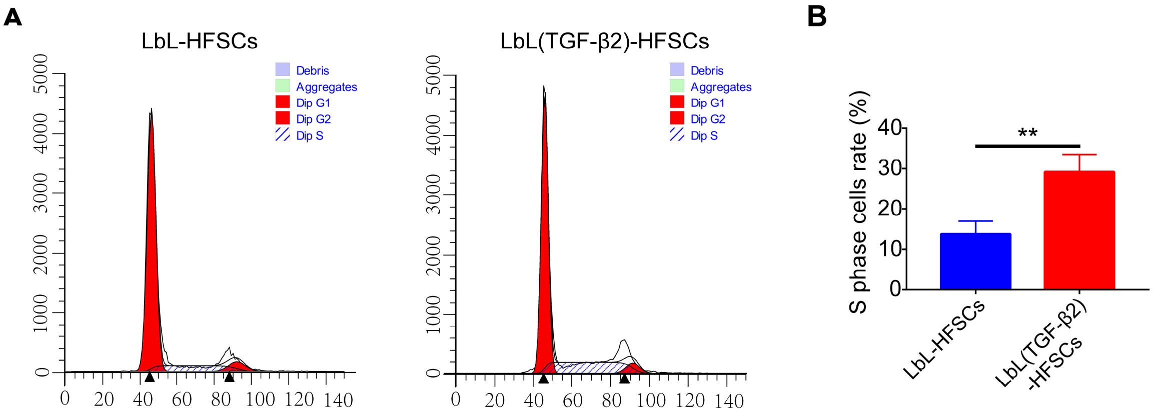

Figure S4. Cell cycle analysis of cell proliferation. (A) A cell cycle kit was used to detect the cell proliferation of layer-by-layer hair follicle stem cells (LbL-HFSCs) and LbL(TGF-β2)-HFSCs cell proliferation after 7 days of culturing. (B) The proportion of S-phase cells for LbL(TGF-β2)-HFSCs was significantly higher than that for LbL-HFSCs. $^{**}p < 0.01$.

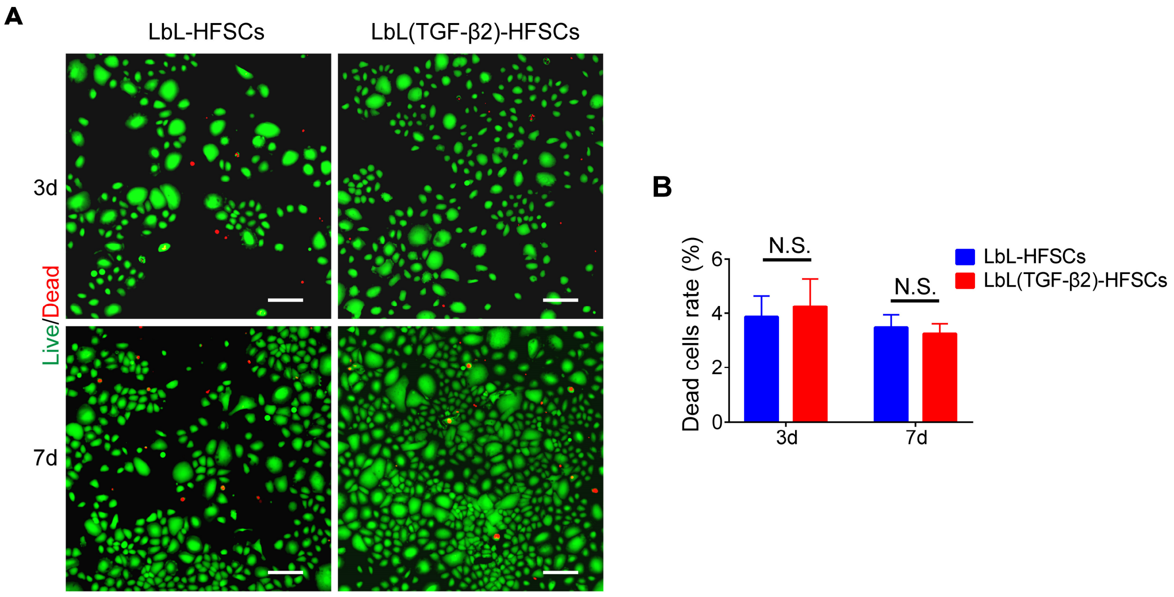

Figure S5. Live/death staining analysis of cell viability. (A) Live/death staining was performed to examine the viability of transforming growth factor (TGF)-β2 unloaded and

loaded layer-by-layer hair follicle stem cells (LbL-HFSCs) after 7 days of culturing. Live (green); Dead (red); Scale bars: 100 µm. (B) There was no significant difference in the proportion of dead cells in the LbL-HFSCs and LbL(TGF-β2)-HFSCs. NS, not significant.

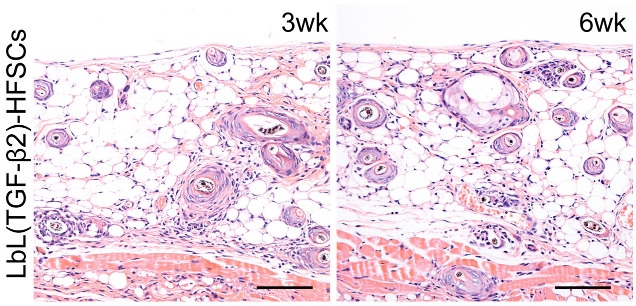

Figure S6. H&E staining to detect the safety of prolonging release of transforming growth factor (TGF)-β2 *in vivo*. After 3 wk or 6 wk of LbL (TGF-β2) -HFSCs transplantation *in vivo*, no tumorigenesis or fibrosis was observed with H&E staining.
